# Supplementary material for: Trends and topics in eye disease research in PubMed from 2010 to 2014
Source: PeerJ. 2016 Jan 7;4:e1557. doi: 10.7717/peerj.1557 (PMC4728026; doi:10.7717/peerj.1557)
Supplement: Supplemental Information 1 — Twenty most frequent check tags retrieved in the study [file peerj-04-1557-s001.docx]

**Supplemental Table S1. Twenty most frequent check tags retrieved in the study**

| Check tag | Number of articles indexed with this MeSH term (%) |
| --- | --- |
| Human | 56 637 (91.17) |
| Male | 36 649 (58.99) |
| Female | 35 878 (57.75) |
| Middle Aged (45-64) | 23 486 (37.81) |
| Adult (19-44) | 20 820 (33.51) |
| Aged (65-79) | 17 600 (28.33) |
| Animals | 9 590 (15.44) |
| Young Adult (19-24) | 9 247 (14.88) |
| Adolescent (13-18) | 8 255 (13.29) |
| Aged, 80 and over | 8 087 (13.02) |
| Child (6-12) | 7 083 (11.4) |
| Child, Preschool (2-5) | 4 446 (7.16) |
| Mice | 3 530 (5.68) |
| Infant (1 to 23 months) | 3 107 (5) |
| Infant, Newborn (to 1 month) | 1 835 (2.95) |
| Rats | 1 821 (2.93) |
| Rabbits | 797 (1.28) |
| Pregnancy | 658 (1.06) |
| Cattle | 223 (0.36) |
| 20th Cent | 211 (0.34) |

Check tags: MeSH terms obligatorily used by indexers to describe recurrent patterns in medical articles in type of material studied (e.g. human, animals, in vitro), gender (male/female), age, chronological elements, and type of study (e.g. comparative study)
