# Supplementary material for: Trends and topics in eye disease research in PubMed from 2010 to 2014
Source: PeerJ. 2016 Jan 7;4:e1557. doi: 10.7717/peerj.1557 (PMC4728026; doi:10.7717/peerj.1557)
Supplement: Supplemental Information 2 — 21st to 50th most frequent MeSH terms located in all the levels beneath the MeSH term “Eye Diseases” in the MeSH tree and co-occurrence of MeSH terms in articles [file peerj-04-1557-s002.docx]

**Supplemental Table S2. 21st to 50th most frequent MeSH terms located in all the levels beneath the MeSH term “Eye Diseases” in the MeSH tree and co-occurrence of MeSH terms in articles**

| MeSH terms located beneath "Eye Diseases" | Number of articles indexed with this MeSH term (%) | Five most co-occurring MeSH terms in articles with the MeSH term in the first column (% of co-occurrence) |  | MeSH terms located beneath "Eye Diseases" (continued) | Number of articles indexed with this MeSH term (%) | Five most co-occurring MeSH terms in articles with the MeSH term in the first column (% of co-occurrence) |
| --- | --- | --- | --- | --- | --- | --- |
| Ocular Hypertension ^(L1)^ | 1005 (1.62) | Intraocular Pressure (74.13)  Tonometry, Ocular (32.64)  Glaucoma, Open-Angle (31.94)  Glaucoma (28.36)  Antihypertensive Agents (24.18) |  | Eye Neoplasms ^(L1)^ | 687 (1.11) | Retrospective Studies (17.47)  Melanoma (16.01)  Treatment Outcome (15.87)  Lacrimal Apparatus Diseases (13.10)  Diagnosis Differential (11.35) |
| Keratoconus ^(L2)^ | 1001 (1.61) | Visual Acuity (44.46)  Corneal Topography (41.06)  Cornea (33.37)  Corneal Stroma (30.67)  Collagen (23.88) |  | Vision, Low ^(L2)^ | 677 (1.09) | Visual Acuity (41.51)  Blindness (30.13)  Visually Impaired Persons (24.96)  Prevalence (18.02)  Questionnaires (16.54) |
| Graves Disease ^(L3)^ | 999 (1.61) | Antithyroid Agents (19.42)  Thyroid Gland (18.72)  Treatment Outcome (14.11)  Thyroidectomy (12.71)  Hashimoto Disease (12.61) |  | Retinal Vein Occlusion ^(L2)^ | 676 (1.09) | Visual Acuity (47.93)  Macular edema (47.34)  Tomography, Optical Coherence (32.54)  Intravitreal Injections (28.99)  Antibodies Monoclonal Humanized (28.11) |
| Astigmatism ^(L2)^ | 947 (1.52) | Visual Acuity (59.03)  Refraction Ocular (42.03)  Myopia (32.73)  Treatment Outcome (29.36)  Corneal Topography(28.41) |  | Eye Abnormalities ^(L1)^ | 671 (1.08) | Mutation (17.88)  Retina (17.44)  Abnormalities Multiple (15.95)  Cerebellar Diseases (14.31)  Kidney Diseases Cystic (13.86) |
| Refractive Errors ^(L1)^ | 943 (1.52) | Visual Acuity (41.99)  Refraction Ocular (28.95)  Retrospective Studies (17.60)  Prevalence (16.33)  Cross-Sectional_Studies (13.36) |  | Retinal Perforations ^(L2)^ | 648 (1.04) | Vitrectomy (57.41)  Visual Acuity (56.33)  Tomography, Optical Coherence (51.23)  Retrospective Studies (39.04)  Retinal Detachment (29.32) |
| Retinitis Pigmentosa ^(L2)^ | 899 (1.45) | Mutation (28.70)  Electroretinography (22.47)  Retina (22.36)  Pedigree (21.91)  Eye Proteins (19.02) |  | Orbital Diseases ^(L1)^ | 647 (1.04) | Tomography, X-Ray Computed (32.61)  Magnetic Resonance Imaging (20.71)  Retrospective Studies (17.00)  Diagnosis Differential (16.23)  Orbit (13.60) |
| Eye Injuries ^(L1)^ | 867 (1.40) | Visual Acuity (21.34)  Retrospective Studies (17.30)  Wounds Nonpenetrating (17.30)  Corneal Injuries (10.5)  Treatment Outcome (10.15) |  | Retinal Neoplasms ^(L2)^ | 634 (1.02) | Retinoblastoma (79.02)  Retrospective Studies (22.08)  Eye Enucleation (17.51)  Treatment Outcome (17.03)  Follow-Up Studies (13.56) |
| Orbital Neoplasms ^(L2)^ | 850 (1.37) | Magnetic Resonance Imaging (28.47)  Tomography, X-Ray Computed (27.76)  Retrospective studies (18.12)  Treatment Outcome (17.06)  Diagnosis Differential (16.82) |  | Diplopia ^(L2)^ | 627 (1.01) | Magnetic Resonance Imaging (25.84)  Tomography, X-Ray Computed (23.76)  Retrospective Studies (20.89)  Treatment Outcome (19.94)  Oculomotor Muscles (19.62) |
| Retinopathy of Prematurity ^(L2)^ | 825 (1.33) | Gestational Age (35.03)  Retrospective Studies (19.76)  Risk Factors (19.52)  Oxygen (16)  Birth Weight (15.39) |  | Uveal Neoplasms ^(L2)^ | 621 (1) | Melanoma (90.18)  Retrospective Studies (18.52)  Prognosis (16.75)  Eye Enucleation (14.81)  Treatment Outcome (14.01) |
| Strabismus ^(L2)^ | 807 (1.3) | Oculomotor Muscles (35.69)  Ophthalmologic Surgical Procedures (25.4)  Visual Acuity (25.15)  Vision Binocular (21.93)  Retrospective_Studies (20.32) |  | Ocular Motility Disorders ^(L1)^ | 593 (0.95) | Magnetic Resonance Imaging (18.72)  Eye Movements (16.19)  Oculomotor Muscles (14.67)  Retrospective Studies (12.98)  Visual Acuity (11.97) |
| Neuromyelitis Optica ^(L3)^ | 766 (1.23) | Aquaporin 4 (45.30)  Autoantibodies (32.25)  Multiple Sclerosis (28.59)  Magnetic Resonance Imaging (25.98)  Immunoglobulin G (18.28) |  | Amblyopia ^(L2)^ | 585 (0.94) | Visual Acuity (48.89)  Strabismus (26.50)  Vision Binocular (17.26)  Treatment Outcome (16.75)  Retrospective Studies (16.24) |
| Eye Infections, Bacterial ^(L2)^ | 741 (1.19) | Anti-Bacterial Agents (44.80)  Endophthalmitis (29.01)  Retrospective Studies (28.21)  Visual Acuity (23.48)  Corneal Ulcer (23.08) |  | Glaucoma, Angle-Closure ^(L3)^ | 580 (0.93) | Intraocular Pressure (51.90)  Gonioscopy (29.31)  Tomography, Optical Coherence (26.21)  Glaucoma Open-Angle (23.28)  Tonometry Ocular (22.24) |
| Eyelid Diseases ^(L1)^ | 737 (1.19) | Eyelids (20.76)  Treatment Outcome (17.91)  Retrospective Studies(17.77)  Meibomian Glands (14.25)  Blepharoplasty (11.80)) |  | Retinal Neovascularization ^(L2)^ | 568 (0.91) | Vascular Endothelial Growth Factor A (37.85)  Disease Models Animal (32.75)  Mice Inbred C57BL (27.82)  Angiogenesis Inhibitors (26.41)  Fluorescein Angiography (26.23) |
| Keratitis ^(L2)^ | 707 (1.14) | Cornea (23.06)  Eye Infections, Fungal (15.13)  Eye Infections, Bacterial (13.30)  Retrospective Studies (12.59)  Antifungal Agents (11.03) |  | Blepharoptosis ^(L2)^ | 567 (0.91) | Oculomotor Muscles (23.63) ;  Blepharoplasty (21.87) ;  Treatment Outcome (21.52);  Eyelids (21.34);  Retrospective Studies (18.52) |
| Endophthalmitis ^(L2)^ | 692 (1.11) | Anti-Bacterial Agents (35.26)  Eye Infections Bacterial (31.07)  Retrospective Studies (25)  Visual Acuity (23.12)  Postoperative Complications (22.98) |  | Wet Macular Degeneration ^(L4)^ | 553 (0.89) | Angiogenesis Inhibitors (58.41) ;  Visual Acuity (57.50) ;  Antibodies Monoclonal Humanized (52.80);  Tomography, Optical Coherence (47.02);  Vascular Endothelial Growth Factor A (46.11) |

^(L1), (L2), (L3), (L4), (L5)^: Levels in the MeSH tree beneath the MeSH term “Eye Diseases”, respectively first, second, third, fourth, and fifth levels
