# Supplementary material for: Trends and topics in eye disease research in PubMed from 2010 to 2014
Source: PeerJ. 2016 Jan 7;4:e1557. doi: 10.7717/peerj.1557 (PMC4728026; doi:10.7717/peerj.1557)
Supplement: Supplemental Information 3 — 21st to 50th most frequent MeSH terms not located beneath the MeSH term “Eye Diseases” in the MeSH tree [file peerj-04-1557-s003.docx]

**Supplemental Table S3. 21st to 50th most frequent MeSH terms not located beneath the MeSH term "Eye Diseases" in the MeSH tree**

| MeSH terms | Number of articles indexed with this MeSH term (%) |  | MeSH terms (continued) | Number of articles indexed with this MeSH term (%) |
| --- | --- | --- | --- | --- |
| Antibodies Monoclonal Humanized | 2 153 (3.47) |  | Mice Inbred C57BL | 1 517 (2.44) |
| Angiogenesis Inhibitors | 2 085 (3.36) |  | Prognosis | 1 492 (2.40) |
| Mutation | 2 072 (3.34) |  | Electroretinography | 1 416 (2.28) |
| Vascular Endothelial Growth Factor A | 2 061 (3.32) |  | Tonometry Ocular | 1 348 (2.17) |
| Disease Progression | 2 048 (3.30) |  | Cataract Extraction | 1 333 (2.15) |
| Intravitreal Injections | 1 963 (3.16) |  | Glucocorticoids | 1 331 (2.14) |
| Tomography X-Ray Computed | 1 908 (3.07) |  | Ophthalmologic Surgical Procedures | 1 331 (2.14) |
| Reproducibility of Results | 1 859 (2.99) |  | Visual Field Tests | 1 322 (2.13) |
| Vitrectomy | 1 824 (2.94) |  | Vitreous Body | 1 290 (2.08) |
| Refraction Ocular | 1 814 (2.92) |  | Genetic Predisposition to Disease | 1 287 (2.07) |
| Questionnaires | 1 760 (2.83) |  | Optic Disk | 1 287 (2.07) |
| Lens Implantation Intraocular | 1 687 (2.72) |  | Phacoemulsification | 1 276 (2.05) |
| Incidence | 1 637 (2.64) |  | Polymorphism Single Nucleotide | 1 263 (2.03) |
| Retinal Ganglion Cells | 1 631 (2.63) |  | Genotype | 1 236 (1.99) |
| Severity of Illness Index | 1 536 (2.47) |  | Phenotype | 1 127 (1.81) |
